# Exploring diabesity pathophysiology through proteomic analysis using *Caenorhabditis elegans*

**DOI:** 10.3389/fendo.2024.1383520

**Published:** 2024-10-30

**Authors:** Malaimegu Subhadra, Dilawar Ahmad Mir, Koley Ankita, Muthukrishnan Sindunathy, Hambram David Kishore, Velayutham Ravichandiran, Krishnaswamy Balamurugan

**Affiliations:** ^1^ Department of Biotechnology, Alagappa University, Karaikudi, Tamil Nadu, India; ^2^ Department of Pharmacology and Toxicology, National Institute of Pharmaceutical Education and Research (NIPER), Kolkata, West Bengal, India

**Keywords:** *C. elegans*, diabesity, triglyceride, lipids, ATGL-1, proteomics, immunofluorescence

## Abstract

**Introduction:**

Diabesity, characterized by obesity-driven Type 2 diabetes mellitus (T2DM), arises from intricate genetic and environmental interplays that induce various metabolic disorders. The systemic lipid and glucose homeostasis is controlled by an intricate cross-talk of internal glucose/insulin and fatty acid molecules to maintain a steady state of internal environment.

**Methods:**

In this study, *Caenorhabditis elegans* were maintained to achieve glucose concentrations resembling the hyperglycemic conditions in diabetic patients to delve into the mechanistic foundations of diabesity. Various assays were conducted to measure intracellular triglyceride levels, lifespan, pharyngeal pumping rate, oxidative stress indicators, locomotor behavior, and dopamine signaling. Proteomic analysis was also performed to identify differentially regulated proteins and dysregulated KEGG pathways, and microscopy and immunofluorescence staining were employed to assess collagen production and anatomical integrity.

**Results:**

Worms raised on diets high in glucose and cholesterol exhibited notably increased intracellular triglyceride levels, a decrease in both mean and maximum lifespan, and reduced pharyngeal pumping. The diabesity condition induced oxidative stress, evident from heightened ROS levels and distinct FT-IR spectroscopy patterns revealing lipid and protein alterations. Furthermore, impaired dopamine signaling and diminished locomotors behavior in diabesity-afflicted worms correlated with reduced motility. Through proteomic analysis, differentially regulated proteins encompassing dysregulated KEGG pathways included insulin signaling, Alzheimer’s disease, and nicotinic acetylcholine receptor signaling pathways were observed. Moreover, diabesity led to decreased collagen production, resulting in anatomical disruptions validated through microscopy and immunofluorescence staining.

**Discussion:**

This underscores the impact of diabesity on cellular components and structural integrity in *C. elegans*, providing insights into diabesity-associated mechanisms.

## Graphical Abstract

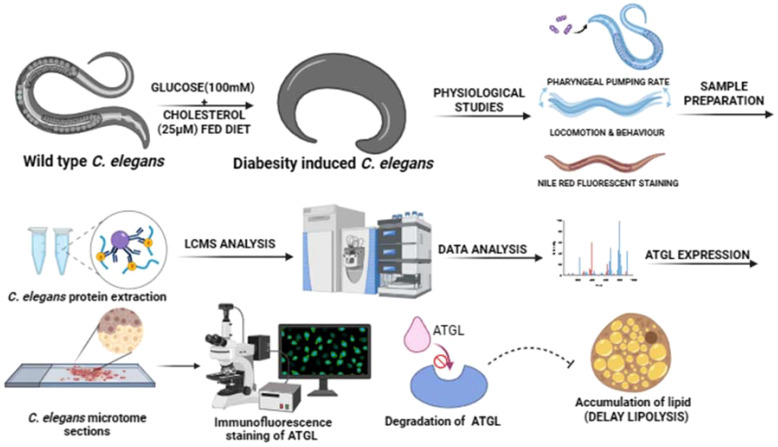

## Introduction

Over the past few decades, there has been a steady rise in the prevalence of overweight and obesity worldwide. Obesity and overweight are significant risk factors for many chronic conditions, such as diabetes, heart disease, and several forms of cancer (1). The combined harmful health impacts of obesity and diabetes are often referred to as “diabesity” (2) to characterize the significant pathophysiological relationship between diabetes and excess body weight. The global obesity pandemic of the past few decades has resulted in an exponential increase in the prevalence of diabetes globally. Additionally, it is anticipated that this may raise the prevalence of other related chronic conditions such dyslipidemia, hypertension, obstructive sleep apnea (OSA), metabolic-associated fatty liver disease (MAFLD), decline brain health and cardiovascular disease (3, 4). The proposed mechanism in the development of diabetes mellitus is visceral obesity, which results in insulin resistance (5). Another theory that has been put out is the overabundance of circulating lipid substrates, like fatty acids, brought on by a high-fat diet, which also causes oxidative stress and chronic inflammation (6, 7). It has also been demonstrated that triglyceride build-up in the liver and skeletal muscle promotes insulin resistance by compromising insulin signaling (8). Studies have elucidated that the metabolic dysregulation due to obesity results in lipotoxicity, cytotoxicity, and adipocyte stress which contribute to the development of diabesity (9). Thus, the most effective way to treat the illness should also involve regulating obesity as much as possible (10, 11). Various studies have shown *C. elegans* to be one of the simplest models for studying fat metabolism and screening anti-obesity agents (12–14).

Recent genome-wide association studies and whole-exome sequencing investigations have uncovered a significant number of genetic variations associated with overweight/obesity and/or T2DM (15, 16). Several model species have been utilized to examine the various factors that can contribute to the onset of diabesity (17–19). In order to comprehend the behavioral and metabolic controls throughout the condition of diabetes, the current study used the model nematode, *C. elegans*. It is the first ever report of diabesity proteomics in this popular model organism. The genetically adaptable organism *C. elegans* has shown to be an excellent model system for novel insights into the regulation of metabolism, aging, animal development, and brain processes. The worm system offers special opportunity to learn about the physiological activities during diabesity condition (12) because the associations between nutrition availability and these physiological processes present several unresolved disease-related biology concerns involving insulin controls.

In the current study, *C. elegans* has been developed as a model to understand the diabesity condition which was confirmed through the estimation of cholesterol, glucose, triglycerides and lipid droplet accumulation after force feeding the high glucose and high cholesterol as diet. The modifications in underlying molecular processes that impact the phenotypes resulting from the condition of diabesity were examined. The impact of diabesity condition on physiology was observed through lifespan, pharyngeal pumping, motility and thrashing assays. Further the investigations have offered additional insights into the potential mechanisms underlying the interaction facilitated by ATGL-catalyzed lipolysis. Altogether, this study delves into ATGL’s role in cell signaling pathways that promote fatty acid and oxidative metabolism in *C. elegans*, particularly in the framework of physiological abnormalities associated with diabesity. Utilizing immunostaining and sectioning techniques on worms reveals unique phenotypes in *C. elegans* under diabesity conditions, compared to control group. Supportively, the proteomics study through LC-MS revealed the molecular mechanisms that underlying pathophysiology of diabesity.

Thus, the present research in *C. elegans* highlighted the understanding on physiological, behavioral and metabolic regulations occurred in connection with diabesity condition. Furthermore, the model, *C. elegans* has continued to be a major contributor in understanding the mechanisms involved in triglyceride accumulation and role of ATGL in fatty acid metabolism through proteomics approach.

## Materials and methods

### Culture conditions

The *C. elegans* N2 Bristol wild-type strain was acquired from the CGC (*Caenorhabditis* Genetics Centre), funded by the NIH National Center for Research Resource in Minnesota, USA. Standard methods as described by Brenner (1974) (20) were utilized for the cultivation and observation of *C. elegans*. N2 worms were cultured and maintained at 20°C on Nematode Growth Media (NGM) plates, utilizing *Escherichia coli* strain OP50 as the primary food source, which served as the control in all assays and analyses. Diabesity in worms was induced by supplementing glucose (100 mM) + cholesterol (25 µM) into nutrient-rich substrate on NGM agar plates. The survival assay was conducted following the protocol outlined by Mir et al. (2020) (21). Synchronized adult-stage organisms were obtained by treating adult-stage hermaphrodites with a mixture of commercial bleach and 5 M KOH in a 1:1 ratio (22).

### Survival assay

Age-synchronized L4 stage worms were subsequently transferred to 24-well plates for three biological replicates containing fluorodeoxyuridine (FUDR). Survival assessments began on the first day of adulthood, with each condition represented by a population of 40 or more worms. *E. coli* OP50 was added to the experimental setup at a cell density of approximately 0.2 OD at 600 nm in the wells. The worms were monitored at 12-hour intervals to record complete survival times. Worms unresponsive to external stimuli such as tapping and gentle touches using a worm picker were classified as dead. Mean and maximum lifespans were calculated using GraphPad Prism, and statistical analyses, including P-values determined using the log-rank (Mantel-Cox) statistic, were performed.

### Pharyngeal pumping assay

To assess the frequency of pharyngeal pumping in both control and diabesity-induced *C. elegans*, a total 25 number of worms per biological replicate were placed onto their respective NGM plates seeded with *E. coli* OP50. The quantification of pharyngeal pumping, measured as the number of contractions per 10 seconds, was observed at specific time intervals using a Stereo microscope, following the method outlined by Durai et al. (2013) (23).

### Motility and thrashing assays

This study utilized N2 nematode strains under two different conditions: Diabesity and standard conditions. The nematodes were cultured on NGM plates containing FUDR regularly. Motility assessments were conducted on the first, fourth, and seventh days of adulthood. Locomotion data were collected and analyzed following the methodology outlined by Rollins et al. (2017) (24). An hour before video recording, the animals were transferred to fresh NGM plates. Young adult to aged synchronized animals were cultured at 20°C for the motility assay. Individual nematodes were tracked, and continuous 1-minute videos were recorded. Wormlab software version 4.1 (MBF Bioscience) (24, 25) was used to analyze the videos. For each strain studied, 10 videos were captured for subsequent analysis. Differences in motility were assessed using the log-rank test in Graph Pad Prism 6.0 software (26–28).

### Nile red staining

To quantify triglyceride accumulation, Nile Red was employed to stain *C. elegans* under both diabesity and standard conditions. Adult worms underwent a series of procedures: initially, they were washed in M9 buffer followed by phosphate-buffered saline (PBS). Subsequently, the worms were fixed in 1% paraformaldehyde at 4°C for 1 hour, following the methodology outlined by Escorcia et al. (2018) (29). Further, the worms were dehydrated using 60% ethanol at room temperature for 15 minutes. The final step involved incubating the worms with a Nile Red staining solution, which consisted of 6 volumes of 1 mg/ml Nile Red in ethanol mixed with 4 volumes of ethanol. This staining process continued for 12 hours as detailed in the protocol by Peng et al. (2016) (30)

### Quantification of Reactive Oxygen Species (ROS)

The ROS was determined utilizing 2’,7’-Dichlorofluorescein diacetate (DCF-DA). Nematodes were collected and suspended in 50 µl of PBS. Subsequently, 10 μl of a 100 mM DCF-DA solution was introduced to the samples, and incubated in darkness for 15 minutes. Following this, samples were washed with M9 buffer. The worms were paralyzed by adding a 1:100 ratios of 100 mM of sodium azide and subsequently the samples were observed and documented using a Nikon fluorescent microscope (31).

### Fourier- Transform Infrared (FTIR) spectroscopy analysis

For protein and fatty acid analysis, an equal number of control and diabesity worms were homogenized with 100 mg of potassium bromide. The resulting mixture underwent vacuum drying to form a solid pellet. Using FTIR an infrared spectrum ranging from 400 to 4000 cm^-1^ was captured. The obtained data points were plotted, represented intensity against wavenumber, following the methodology detailed by Sethupathy et al. (2017) (32).

### Quantification of glucose levels using the glucose oxidase- peroxidase method

To ascertain the glucose concentration, the worms were homogenized using the Mini homogenizer MT-13K and centrifuged at 7,500 rpm for 5 minutes to remove the debris. An equivalent concentration of sample was mixed with 1000 μl of Autospan Liquid Gold Glucose reagent, facilitating the quantification of glucose levels through the enzymatic oxidation of glucose. This oxidation process yields gluconic acid and hydrogen peroxide as products. Following a 10-minute incubation period, the color change resulting from the hydrogen peroxide reaction was evaluated using a chromogen. The assessment was performed using a Semi auto analyzer, measuring absorbance at a wavelength of 505 nm (33).

### Estimation of triglyceride levels by GPO method

To evaluate the triglyceride level, an equal concentration of sample was mixed with 1000 μl of triglyceride reagent Liqui CHECK™. The enzymatic measurement of triglycerides involved a calorimetric quantification process, where the reaction’s intensity was measured at 546/630 nm wavelengths. This assessment was performed using a Semi Auto Analyser system following the methodology outlined by Sullivan et al. (1985) (34).

### Isolation of lipids using Methyl-tert-butyl-ether method

Approximately 50 x 10^5^ worms were used to extract lipids from worms. For each 200 μl sample aliquot, 1.5 ml of methanol was added into glass tubes and vigorously vortexed. Subsequently, 5 ml of MTBE (methyl tert-butyl ether) was added to the mixture, and incubated for one hour incubation at room temperature. Following this, 1.25 ml of MS-grade water was added to the mixture to induce phase separation. The mixture was incubated for 10 minutes at room temperature and then centrifuged at 1,000 g for 10 minutes. The upper (organic) phase was collected, while the lower phase underwent re-extraction as described by Matyash et al., 2008 (35).

### Thin layer chromatography analysis

The lipid extract analysis using Thin-Layer Chromatography (TLC) was conducted on Merck Silica Gel 60 HPTLC plates measuring 10 × 10 cm. The development of silica gel plates was prepared using a solvent mixture comprising n-hexane, diethyl ether, and acetic acid in a ratio of 75:13:9 (v/v/v). To visualize the lipid bands, a spray solution consisting of 20% H_2_SO_4_ in ethanol was applied. Subsequently, the plates were charred at a temperature of 200°C to visualize the lipid bands, following the method described by Touchstone et al. (1995) (36).

### Immunofluorescence staining

Tissue sections from frozen blocks of both control and diabesity samples were cut at a thickness ranging from 2 to 4 μm to ensure optimal resolution and placed on microscope slides. Subsequently, 10% neutral buffered formalin (NBF) was applied onto the slides and allowed to incubate for approximately 10 to 15 minutes. Heating at 58°C for one hour was conducted for proper preparation of the sections. Following this, the sections underwent deparaffinization and hydration steps. The immunohistochemical process was initiated by fixing the sections with a 4% v/v paraformaldehyde solution in 1X PBS at room temperature. After fixation, the sections were washed twice and then permeabilized using a 0.2% Triton X-100 solution in 1X PBS for 20 minutes at room temperature. Subsequent to permeabilization, the sections were blocked using 2% BSA. After another round of washing, the sections were left to incubate overnight with a primary antibody at a dilution of 1:50, maintained at 4°C. Following incubation with the primary antibody, the sections underwent three washes with PBS and were then incubated with secondary antibodies labeled with Alexa Fluor 488 (Invitrogen) for 1 hour at room temperature. A washing step using diluted 1% BSA in 1X PBS for 5 minutes followed this incubation. Digital images were acquired using a fluorescence microscope from Nikon, Japan, and the process was facilitated using Nis-Elements D software (37)

### LC-MS analysis

The LC-MS analysis was conducted based on the methodology outlined in (38), with minor adjustments. Initially 200 mM freshly prepared DTT was combined with 200 µg of protein per sample in a volume of 100 µl. This mixture was incubated in darkness for 1 hour at room temperature. Subsequently, 20 µl of 200 mM IAA was added and incubated for 1 hour at a temperature of 56°C. Following the addition of 200 mM DTT, the sample underwent an in-solution trypsin digestion using trypsin in 55 mM acetic acid at a 1:50 ratio. The samples were then left to incubate for 16 hours at 37°C. For the subsequent LC-MS analysis, an Agilent 6545 ESI-LC/qToF-MS/MS instrument was utilized. Data acquisition was performed using Mass Hunter Workstation software, version B.08.00, while data processing employed Spectrum Mill software, version 06.00. Regarding taxonomic assignment, *C. elegans* was designated as the organism of interest. A mass tolerance of 20 ppm was set for the precursor mass, while the product mass tolerance was established at 600 mDa (39).

### Protein data analysis

To identify shared proteins within control and diabesity samples protein identification from the LC-MS analysis was subjected to the Venny 2.1.0 tool *(http://bioinfogp.cnb.csic.es/tools/venny/)*, an online platform for creating Venn diagrams. The Gene Ontology (GO) classification of the proteins identified through mass spectrometry was performed using the UniProt database *(http://www.uniprot.org/uploadlist/)* in accordance with the methodology outlined in (38). To explore the interactions among the identified proteins, the STRING Version 10.5 online tool *(http://string-db.org/)* was utilized. A medium confidence score of 0.400 was employed to assess protein-protein interactions, as described by Balasubramaniam et al. (2020) (39). Furthermore, the pathways regulated by the identified proteins and gene enrichment analyses were carried out using the KEGG Mapper - search pathway online tool *(http://www.genome.jp/kegg/tool/map_pathway1.html)*. This tool, as described by Pooranachithra et al. (2021) (40), allowed for the exploration of pathways and gene enrichment associated with the identified proteins.

### Statistical analysis

All the experiments were performed independently in triplicates. Results are represented as mean ± standard errors of the mean (SEM). The significance of statistical difference was analyzed using one-way ANOVA with Dunnett’s multiple comparisons test (*p < 0.05, **p < 0.01, ***p < 0.001, ****p < 0.0001) in SPSS software version 27.0.1 and GraphPad Prism 9 (41).

## Results

### Diabesity decreased *C. elegans*, lifespan and pharyngeal pumping

**Figure 1:**
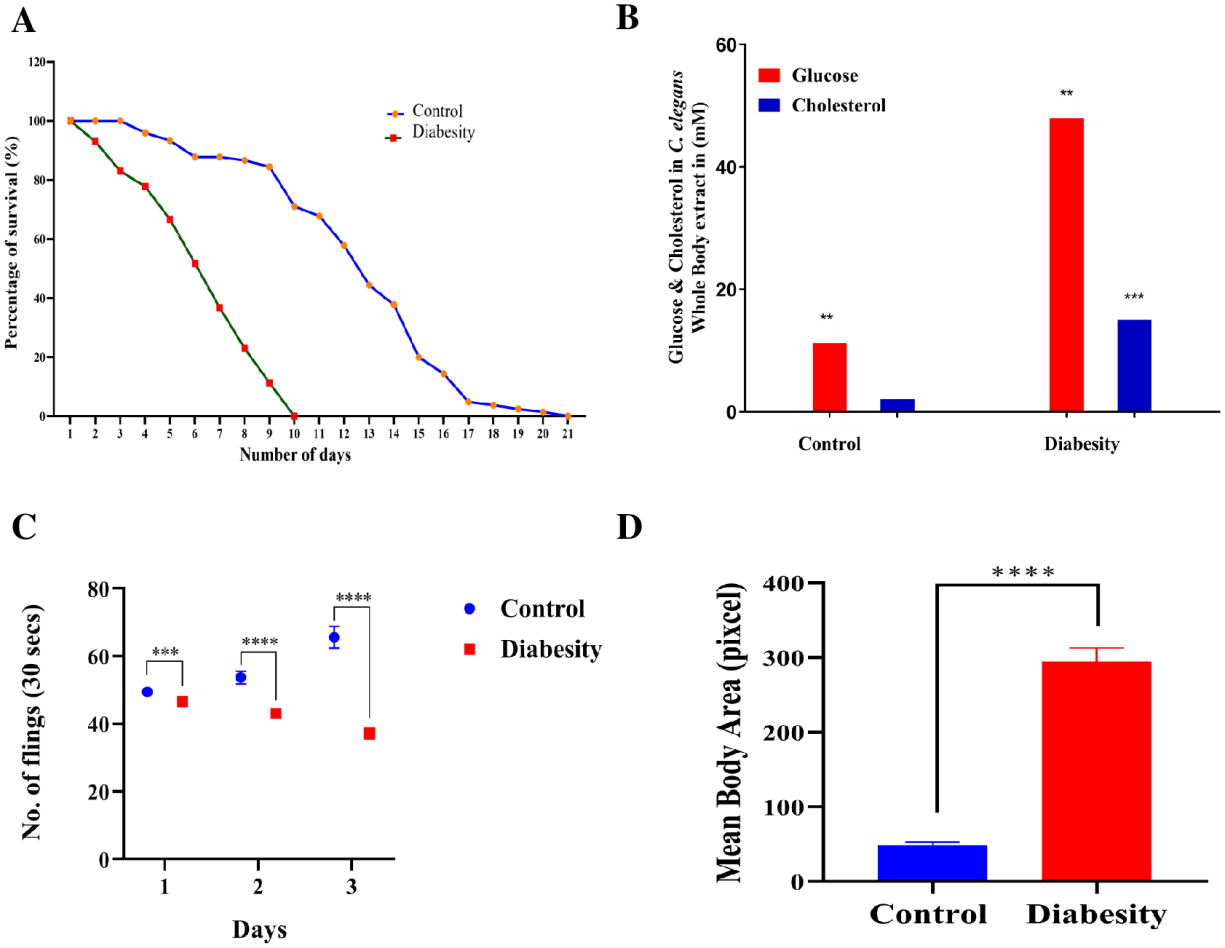
Physiological assays demonstrating the impact of high glucose and cholesterol on *C. elegans* life span. **(A)** Survival assays revealed a significant decrease in *C. elegans* lifespan under diabesity conditions compared to the control with *E. coli* OP50. Kaplan-Meier survival curves were compared using the Mantel-Cox log-rank test. The maximum reduction in lifespan was observed at 60% compared to controls (P < 0.0001, n = 160). **(B)** *C. elega*ns cultured on agar supplemented with 100 mM glucose and 25 μM cholesterol showed altered median lifespan (day 5th of adulthood), and concentrations of glucose and cholesterol were quantified in whole-body extracts at this stage. Results represent the means of three biological experiments and were assessed using a t-test (*P > 0.05, **P < 0.001, and ***P < 0.0001) to compare the two groups. **(C)** Pharyngeal pumping of *C. elegans* significantly decreased under diabesity conditions from day 2 of adulthood. The experiment was conducted in triplicate. Results are from a representative experiment out of three biological experiments, each involving more than 80 nematodes. *Statistical differences were considered significant at p < 0.05 **(D)** Body size of *C. elegans* significantly increased under diabesity conditions.

To understand the mechanistic basis of diabesity, *C. elegans* were raised in NGM containing high glucose and cholesterol rich diet (see Methods). It has been reported that growing *C. elegans* on NGM plates containing 40 mM glucose resulted in an intracellular concentration of 14 mM, a range observed in poorly controlled diabetic patients (12, 14). In order to understand the impact of diabesity condition generated by high glucose and cholesterol-rich diets in *C. elegans*, the changes were observed in lifespan. The introduction of high glucose and cholesterol-rich diets resulted in a significant reduction in both mean lifespan—from 10 ± 0.6 to 5.5 ± 0.4 days (P < 0.0001)—and maximum lifespan—from 21 ± 0.4 to 10 ± 0.4 days (P < 0.0001)—when compared to the control group fed with *E. coli* OP50 (**Figure 1A**).

For the analysis of intracellular glucose and cholesterol concentrations at the mean lifespan (day 5 of adulthood), whole-body extracts from *C. elegans* were examined. Culturing *C. elegans* on agar plates containing 100 mM glucose and 25 μM cholesterol under diabesity conditions resulted in intracellular concentrations of 48 mM glucose and 15 μM cholesterol (P < 0.001, n=50), notably higher than the control worms’ levels at 11 ± 4 mM glucose and 2 ± 1 μM cholesterol (P < 0.0001, n=50) (**Figure 1B**).Under standard conditions, no supplementary glucose is introduced into the nematode growth medium. However, in all subsequent experiments, the high glucose and cholesterol conditions for *C. elegans* refer to concentrations of 100 mM glucose and 25 μM cholesterol, respectively. This distinction was crucial in assessing the consequences of diabesity conditions induced by these dietary components in *C. elegans*. In addition, when compared to the control group, the pharyngeal pumping of diabesity *C. elegans* progressively decreases starting from day 2 of adulthood (**Figure 1C**). After day 6^th^ of adulthood, all other physiological activities also progressively decreased until *C. elegans* becomes immobile. Compared to the control, diabesity worms exhibit other defective physiological characteristics such as a fattened body and apathetic behavior (**Figure 1D**). Each experiment was conducted in biological triplicate, and the error bars represent the mean ± SEM (p < 0.005).

### Nile red staining showed high lipid species deposition in diabesity worms

**Figure 2:**
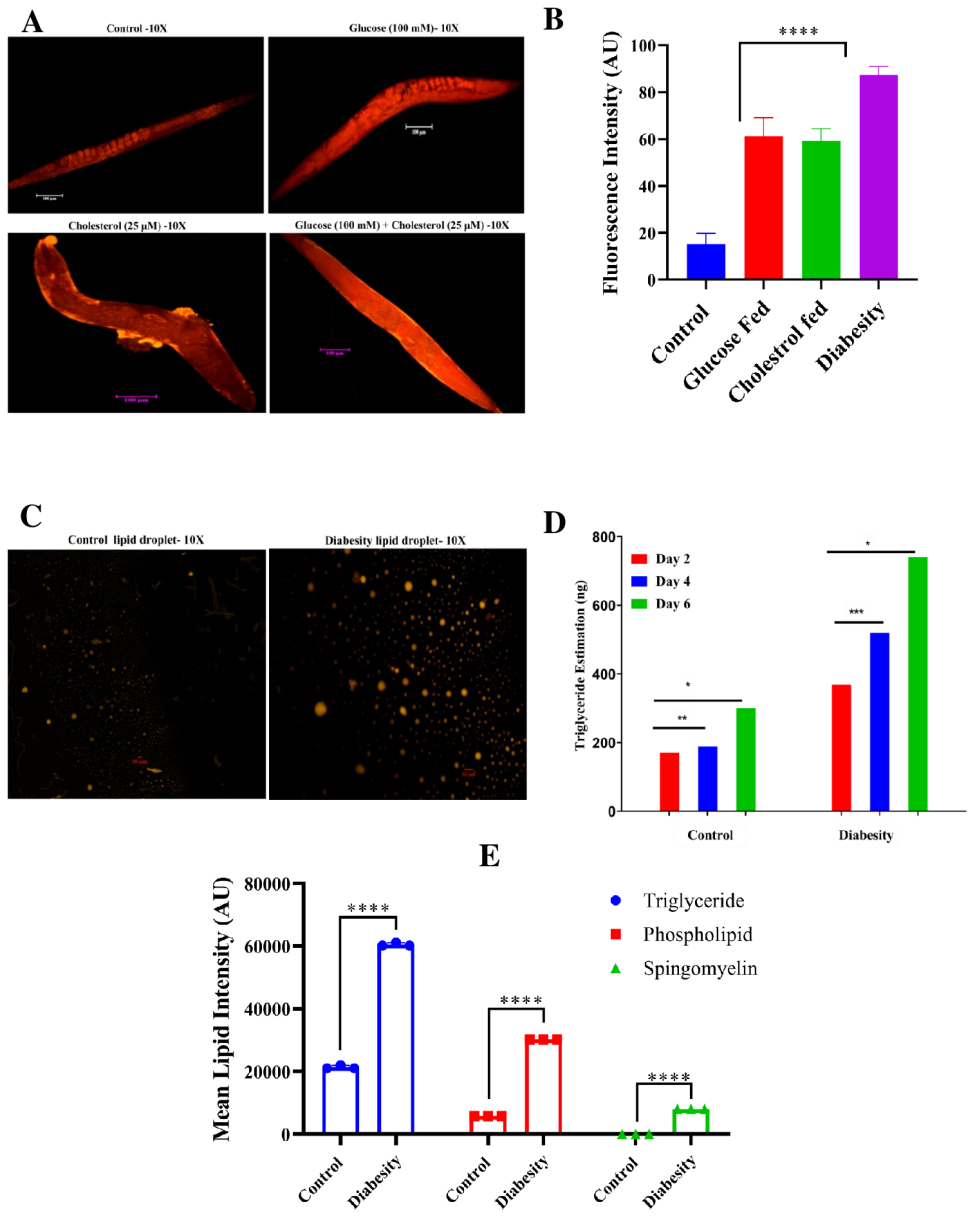
Analysis of *C. elegans* lipid accumulation using Nile red staining. **(A)** A representative fluorescent micrograph of Nile red staining depicts the distribution and accumulation of lipid droplets in *C. elegans* under diabesity and control conditions. **(B)** Quantitative analysis of Nile red staining using ImageJ software, confirms that compared to control and other conditions, diabesity-induced animals exhibit higher lipid deposition. **(C)** A representative fluorescent micrograph of Nile red staining depicts the isolated lipid droplets from *C. elegans* under diabesity and control conditions. **(D)** Estimation of triglycerides shows they are significantly high in diabesity animals compared to control (P< 0.0001). **(E)** A quantitative analysis of thin-layer chromatography confirms that, in comparison to the control group, animals under diabesity conditions exhibit higher percentages of triglycerides, cholesterol esters, sphingomyelin, and phospholipids compared to control (P< 0.0001).

Due to the absence of adipocytes, *C. elegans* stores its fat in small droplet-like organelles referred to as lipid droplets, predominantly located in the intestine and hypodermis (42). To investigate the impact of high glucose and cholesterol on lipid droplets accumulation in *C. elegans*, Nile red staining was conducted. This staining method targets neutral lipids such as triglycerides or cholesterol esters. The fluorescence from Nile red staining was quantified using ImageJ software. The supplementation of high glucose to the NGM resulted in a significant increase in lipid accumulation in diabesity samples compared to the control (**Figures 2A, B**), consistent with previous findings that used glucose as an obesogenic stimulus in *C. elegans* (43, 44). Intracellular fat droplets in *C. elegans* were visualized using fluorescence microscopy (45), and the present study revealed a notable increase in fat and lipid storage within adipose tissues in the diabesity condition and no significant fluorescence was observed in control (**Figure 2C**).

Furthermore, the quantification of triglycerides indicated a substantial elevation in diabesity samples compared to the control. Triglyceride levels were assessed at day 2, 4, and 6 of adulthood. At day 2 of adulthood, diabesity animals exhibited approximately 369 ± 1 ng of triglycerides compared to 170 ± 3 ng in the control (P< 0.0001). By day 4 of adulthood, diabesity animals showed 520 ± 5 ng compared to 188 ± 5 ng in the control (P< 0.0001, n=500). Finally, at day 6 of adulthood, diabesity animals displayed 740 ± 1 ng of triglycerides, whereas the control exhibited 300 ng (P< 0.001, n=500) (**Figure 2D**). In this study, we have also conducted the separation of lipid extracts through thin-layer chromatography following the standard procedure (see methods). Our results demonstrate a significant increase of triglycerides, cholesterol esters, and notably higher amounts of glycolipid species (Sphingomyelin and Phospholipids) in diabesity animals compared to the control group (**Supplementary Figure S1**). The proportional content analysis from each lane of the TLC plates was performed by densitometry of digital photographs using ImageJ software (**Figure 2E**). Each experiment was conducted in biological triplicates, and the error bars represent the mean ± SEM (p < 0.001).

### Diabesity induced oxidative stress alter cellular components (lipids and proteins)

**Figure 3:**
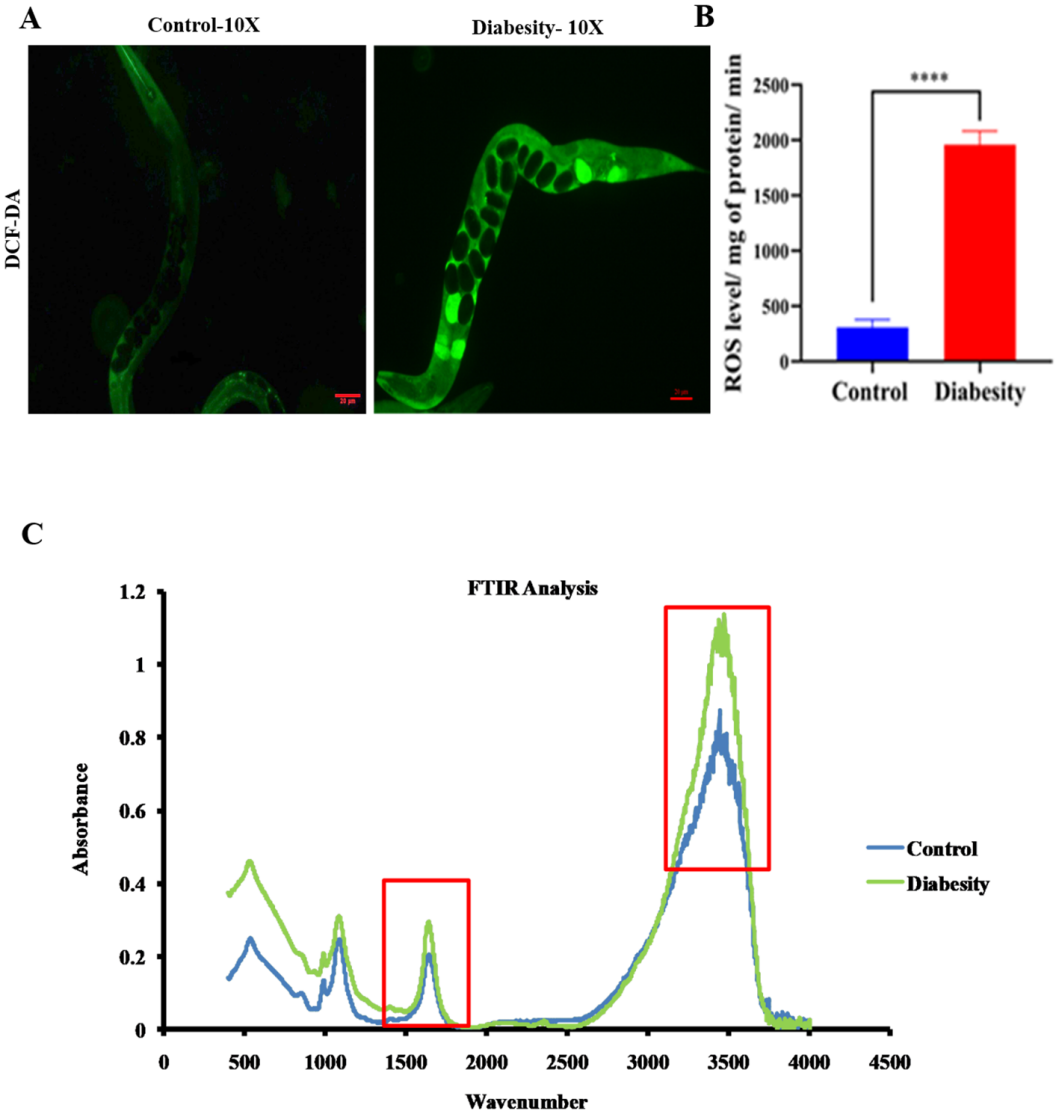
Diabesity animals exhibit elevated levels of ROS production and impacting cellular components (Lipids and proteins). **(A)** Assessment of ROS levels using H_2_DCFDA staining on day 5th of adulthood demonstrates the percentage of ROS generation in diabesity worms through micrograph analysis. **(B)** Quantitative analysis of the ROS micrographs using ImageJ software. Shown are animals from a representative experiment out of 4 biological independent experiments, each including 30 worms **(C)** FT-IR analysis of *C. elegans* lipids and proteins. The average FT-IR spectra of the diabesity showed major signature peak difference at 3,000–2,800 cm^−1^, 1800-1500 cm^−1^ and 1200 -900 cm^−1^, these regions belong to fatty acids, carbohydrate bands, carbonyl stretching and vibration of the lipids respectively. Data were expressed as mean value of three experiments and the error bars represent SEM ± mean (*p < 0.05).

Lipid peroxidation has been observed to be higher in obese individual mice compared to non-obese (46, 47). Hyperglycemia results in elevated levels of reactive oxygen species (ROS) production through the polyol pathway, leading to the formation of advanced glycation end products (AGEs) and causing significant oxidative stress (48). Assessing ROS levels serves as an effective means to quantify oxidative damage resulting from hyperglycemia. In this study, we measured ROS levels using H_2_DCFDA staining. ROS induction was examined at day 5^th^ of adulthood. We found a higher generation of ROS in worms exposed to diabesity conditions, whereas no significant fluorescence was observed in the control sample (**Figures 3A, B**).

The application of FT-IR spectroscopy has proven to be a valuable tool for investigating biomolecular complex structures within cells (49). In the context of examining the impact of oxidative stress induced by diabesity on *C. elegans* lipids and proteins, FT-IR analysis revealed significant differences in intensity peaks between diabesity samples and the control group. Specifically, the analysis of *C. elegans’* fatty acids exhibited distinct intensity peaks at various wavelengths: 3000-2800 cm^-1^, 3100 cm^-1^, and 2930 cm^-1^. These peaks correspond to fatty acids, N-H stretching of proteins associated with lipids, and CH_2_ anti-symmetric stretching of lipids, respectively (50–52) (**Figure 3C**). Additionally, the protein FT-IR spectroscopy analysis depicted features such as carbonyl stretching (1800 - 1500 cm^-1^), vibration of Amide I and Amide II bands of proteins (1740 cm^-1^), and broadening of the peak at 1650 cm^-1^, suggesting protein oxidation (53–55). Furthermore, noticeable differences in intensity were observed in the 1200-900 cm^-1^ range, associated with carbohydrate bands (56–58). These spectral differences in the *C. elegans* fatty acid and protein region imply the impact of stress induced by high diabesity conditions on cellular molecules. Moreover, differences in protein FT-IR spectroscopy indicate variations in protein glycosylation. Changes in protein glycosylation have been linked to various physiological and pathological conditions, such as tumor invasion, cell growth differentiation, host-pathogen interaction, cell trafficking, and transmembrane signaling (59).

### Diabesity condition reduced locomotion in *C. elegans*

**Figure 4:**
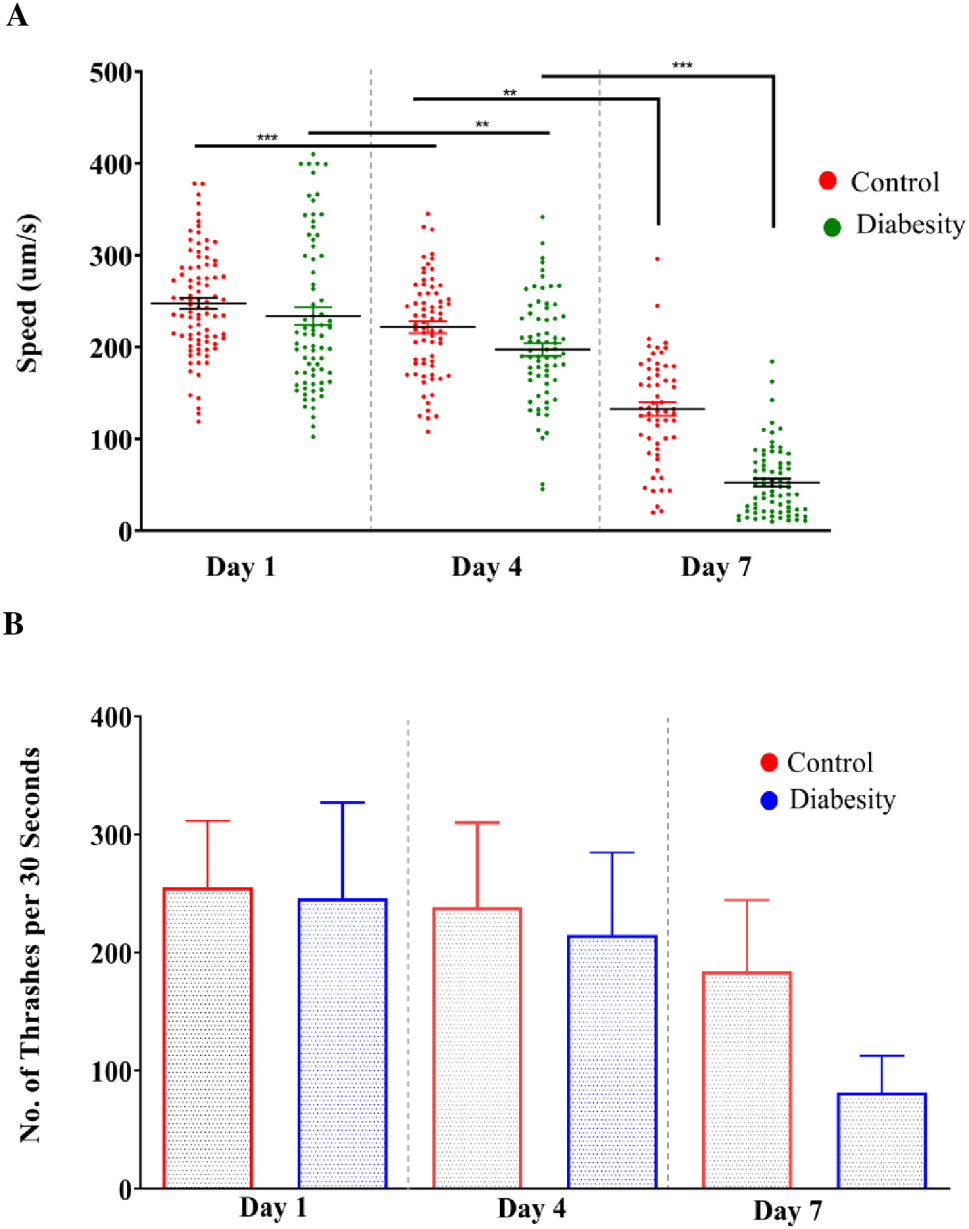
Evaluation of motility and thrashing of diabesity and control worms: The motility of *C. elegans* treated with high glucose and cholesterol was assessed while analyzing the video recording (See Methods). Speed and thrashing moments was measurements were at three different time points (day 1, 4th, and 7th day) of adulthood. (**A, B**) Motility and Thrashing moments of diabesity and control worm respectively. For each experiment, over ~40 worms were tracked for 60 seconds. Scatter plots depict bars representing the mean and standard error of the mean for all conditions. Statistical comparisons were conducted using one-way ANOVA with Dunnett’s multiple comparisons test (*p < 0.05, **p < 0.01, ***p < 0.001, ****p < 0.0001)).

High glucose causes susceptible to cognitive impairment, progression of Alzheimer’s disease and decreases the locomotor activity (60–64). The motility of *C. elegans* is regulated by acetylcholine receptors, which plays important role in synaptic functions (65). The ability to move relies on neuronal signaling, maintenance of muscle mass, and integrity of connective tissue (66–69). Consequently, motility serves as a valuable indicator for evaluating the neurotransmission and is a useful marker to assess health span (70). The motility assays conducted in our study were devised to investigate the intricate connections between high glucose and locomotor behavior. Our study involved the examination of changes in motility at two distinct time points: day-3 and day-6 of adulthood. We conducted these assessments in both under standard conditions and diabesity condition. We utilized MATLAB worm tracking software to analyze quantitative data obtained from video recordings of motility (see Methods). At both day 3 and day 6 of adulthood, nematodes experiencing the diabesity condition displayed decreased motility compared to those in standard conditions (**Figure 4A**). Additionally, worms under standard conditions displayed a notably higher frequency of thrashing movements than those in the diabesity condition (**Figure 4B**). The dopamine signaling in *C. elegans* is responsible for the gait transition from swim to crawl (71). Studies using ontogenetic confirmed D1-like dopamine receptors switch animals, away from the bacterial food source (72–74). In our studies diabesity condition, nematodes move off from the food source *E. coli* OP50 bacteria, compared to those under standard conditions (data not shown).

### Proteomic analysis of diabesity model

**Figure 5:**
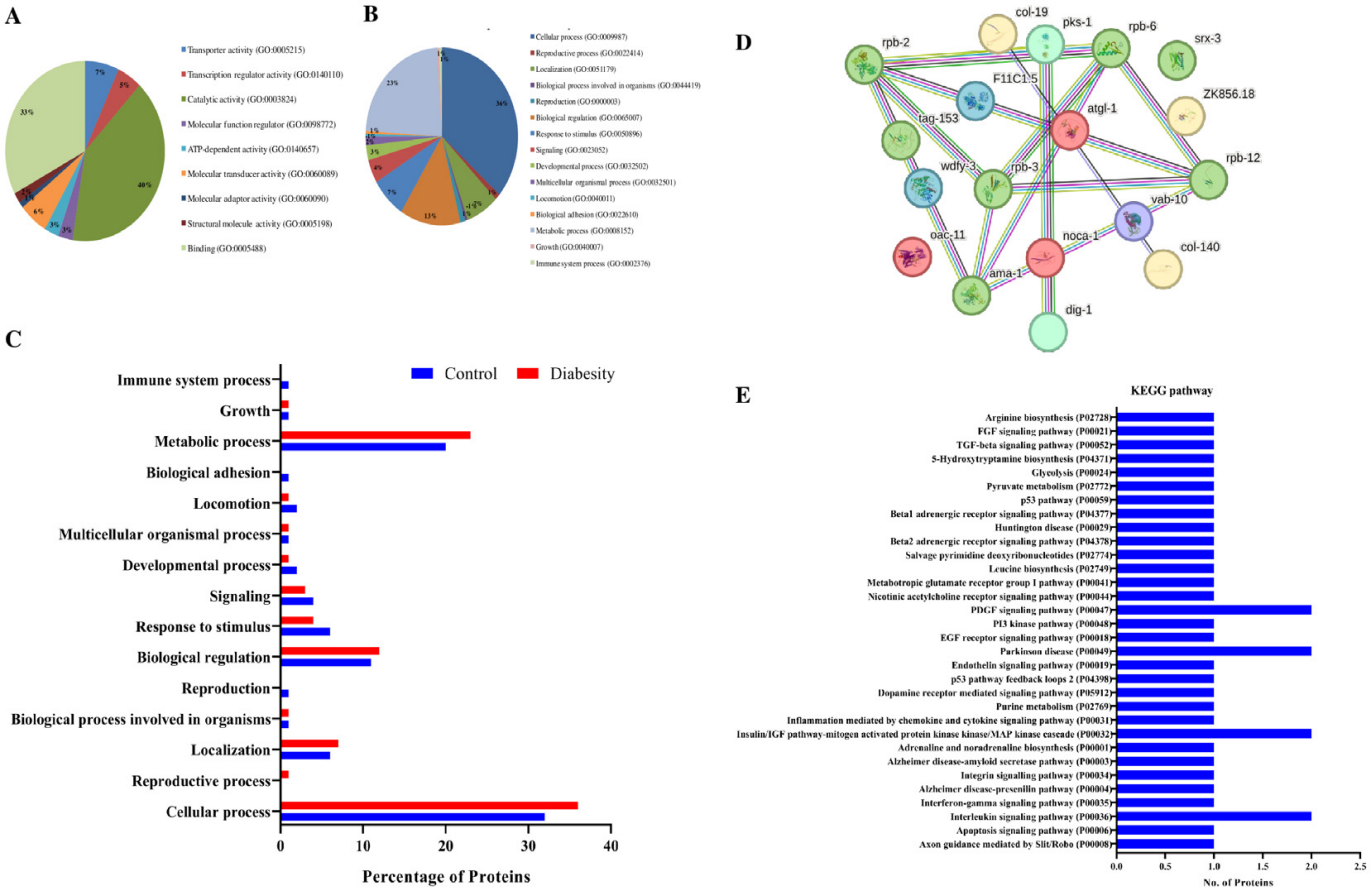
Bioinformatics analysis of Proteomics Data. **(A, B)** Gene ontology analysis using the UniProtKB online tool showed regulated proteins are involved in cellular processes, locomotion, immune system processes, responses to stimuli, and signaling pathways. **(C)** Functional annotations and protein enrichment scores of regulated proteins was performed using the DAVID tool. The biological functions which have more number of regulated proteins binding, catalytic activity, and transport activity. **(D)** Interactome map among regulated proteins was performed using STRING tool with a median confidence threshold score (0.400). The interaction map showed link between identified regulated proteins of *C. elegans*. The regulated proteins showed interaction among Toll-like receptor (TLR) activation, plasma lipoprotein remodeling, serine biosynthesis, LDL clearance, lipid transporter activity, and unblocking of NMDA receptors. **(E)** KEGG pathway analysis shows regulated proteins were predominantly associated with pathways such as the insulin/IGF pathway, Alzheimer’s disease pathway, and nicotinic acetylcholine receptor signaling pathways.

The results obtained from physiological assays prompted further investigation into the primary molecular mechanisms underlying *C. elegans* diabesity. LC-MS-based proteomic analyses were conducted in biological triplicates to ensure the robustness and reproducibility of the results. Following Post-MS Data analysis for protein identification and expression, a total of 627 differentially regulated proteins were detected. We set the relative expression ratio for downregulated and upregulated proteins between control and treated samples at ≥-1.5 and ≥1.5, respectively, across all biological replicates (p < 0.05). Out of these, 18 proteins were common with 10 being down regulated and 8 being up regulated significantly. Detailed information on these differentially regulated proteins can be found in **Supplementary Tables S1**, **S2**. Subsequently, the high-throughput protein profile and expression data underwent comprehensive bioinformatics analysis. To assign potential physiological functions to the regulated proteins, a Gene Ontology (GO) annotation and functional enrichment analysis were performed using the UniProtKB tool. The differentially regulated proteins were categorized into molecular functions, biological processes, and cellular components. Based on the GO functional annotation, the major group of regulated proteins in diabesity is associated with response to stimuli and cellular processes. Additionally, genes related to locomotion, localization, immune system processes, responses to stimuli, and signaling pathways exhibit regulatory changes. The primary biological processes predominantly affected among the differentially regulated proteins include metabolic processes and cellular activities (**Figures 5A, B**).

The functional annotation and gene enrichment of all regulated proteins were conducted using the DAVID tool. Functional annotation delineates the role of individual proteins across various biological processes. Among the biological functions exhibiting the highest numbers of differentially regulated proteins between the control and diabesity conditions were binding and catalytic processes. Notably, transporter activity genes showed an upregulation in diabesity compared to the control, as illustrated in (**Figure 5C**). In diabesity, proteins responsible for signaling receptor activity, nutrient reservoir activity, fusogenic activity, cytoskeletal motor activity, and transcription coactivator activity were observed to be downregulated when compared to the control.

The interactome network was constructed using the STRING tool to analyze regulated proteins, investigating their interactions and predicting functional associations. The resulting interaction map of regulated proteins illustrates the relationships among molecular players involved in Toll-like receptor (TLR) activation by endogenous ligands, plasma lipoprotein remodeling, serine biosynthesis, LDL clearance, lipid transporter activity, and unblocking of NMDA receptors (**Figure 5D**). These proteins are interconnected, either directly interacting with one another or indirectly through their partners. Moreover, employing the STRING tool helped in identifying potential dysregulated KEGG pathways associated with diabesity. Subsequently, KEGG pathway analysis unveiled that differentially regulated proteins were predominantly associated with pathways such as the insulin/IGF pathway, Alzheimer’s disease pathway, nicotinic acetylcholine receptor signaling pathways, and the P53 pathway. The pathways most significantly regulated in diabesity are depicted in (**Figure 5E**).

### Diabesity decreases collagen production and disruptions anatomical structures

In mice lacking collagen, the development of insulin resistance and glucose intolerance occurs, accompanied by elevated serum triglycerides and fat accumulation (75, 76). In *C. elegans*, deficiency in ATGL-1 results in elevated lipid content and shortened lifespan. Despite ATGL-1 being identified as a cytosolic lipase, nuclear morphology deterioration is not rescued upon upregulation of ATGL-1 (77). In our studies, we observed a noteworthy increase in lipid droplets and triglycerides within diabesity models (**Figure 2**). The accumulation of triglycerides during diabesity is linked to ATGL-1 deficiency, preventing their hydrolysis and leading to their encapsulation in lipid droplets. Consequently, organelles undergo enlargement due to this accumulation.

**Figure 6:**
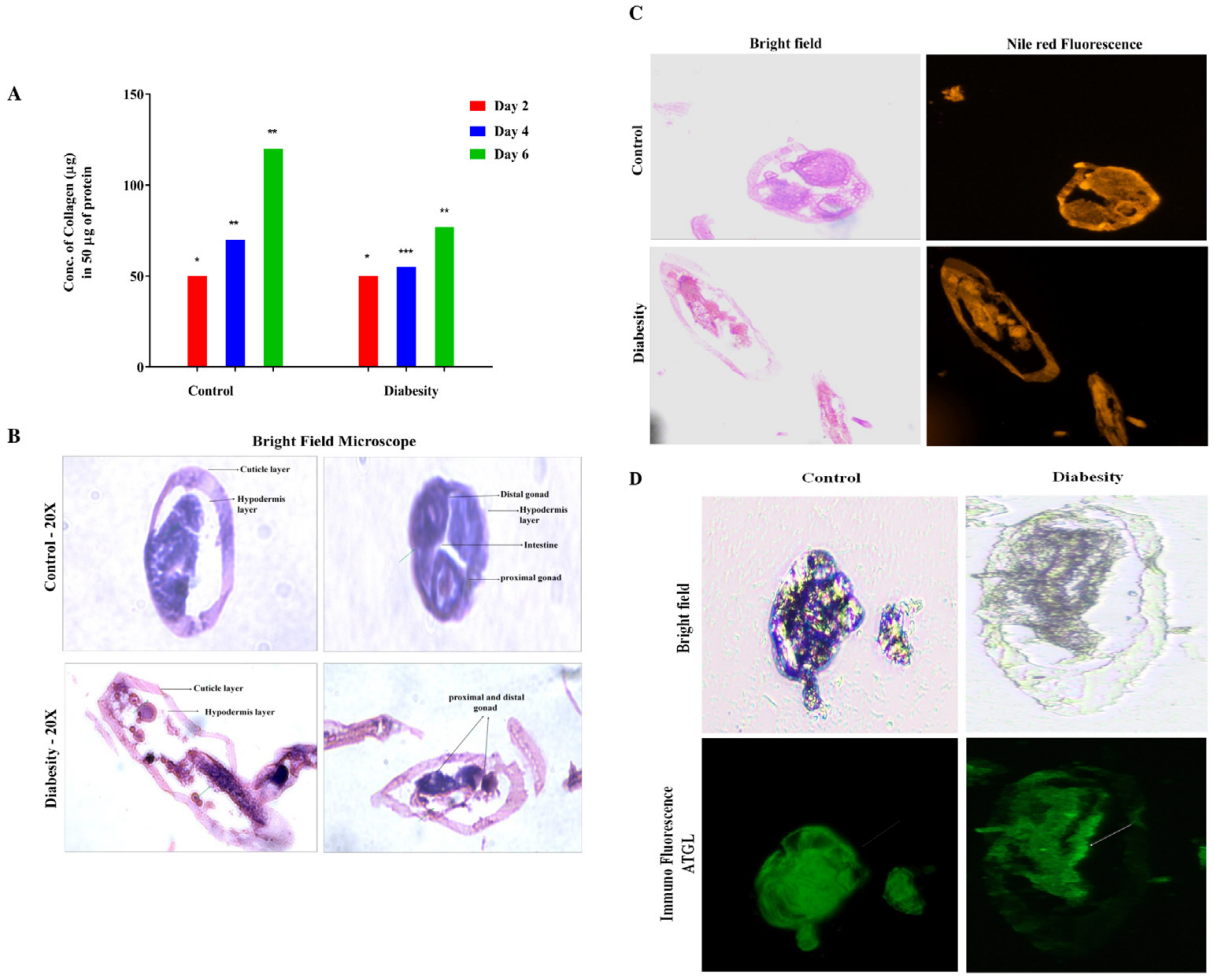
Impact of diabesity on collagen and worm body structures. **(A)** Estimation of total collagen were measured at three distinct time points (day 2, 4^th^, and 6^th^ day) during adulthood using a total of over 10,000 worms per experiment. The results present the means derived from three separate biological experiments and were statistically analyzed via a t-test (*p < 0.05, **p < 0.01, ***p < 0.001, ****p < 0.0001) to compare diabesity and control groups. **(B)** The *C. elegans* transverse section image depicts tissue structure differences within diabesity and control worms; Scale bar- 100μm **(C)** The *C. elegans* transverse sections under bright field and stained with Nile red to observe the lipid droplet distribution in diabesity condition compared to the control groups; Scale bar- 50μm. **(D)** Tissue sections represent the overall distribution of ATGL in control and diabesity condition using ALEXA fluor 488. The green color delineates the presence of ATGL along with structural differences, showing notable changes in specific aspects of the worm’s body structure. The white arrows represent the presence of ATGL in diabesity compared to control groups; Scale bar- 100μm.

In this study, one of the focuses was isolation and estimation of *C. elegans* collagen (see methods). Our investigation into diabesity revealed a significant reduction in collagen levels at the 4^th^ and 6^th^ days of adulthood compared to the control group. Specifically, control specimens exhibited 80 ng/50 mg (4^th^ day) and 120 ng/50 mg (6^th^ day), while diabesity-afflicted animals demonstrated 50 ng/50 mg and 70 ng/50 mg respectively (**Figure 6A**). Notably, no substantial changes were observed at day 2 of adulthood. These findings emphasize the influence of diabesity on collagen production within the *C. elegans* model, indicating a potential interplay between metabolic disorders and collagen regulation. The reduced collagen levels could lead to disruptions in anatomical structure and its integrity, due to sustained elevated levels of glucose and triglycerides. To validate these abnormalities, our microscopic analysis of control sections exhibited well-defined epidermal and cuticle layers, including specialized worm body structures such as the hemidesmosomes layer. Conversely, in diabesity, these specialized features like cuticles and hemidesmosomes were less distinct and displayed compromised mechanical strength (**Figures 6B, C**). Immunofluorescence staining was conducted to visualize the distribution and subcellular localization of ATGL-1 (see Methods). The results revealed a predominant presence of ATGL-1 in the epidermal region and hemidesmosomes in the control group. Conversely, in diabesity-induced conditions, its presence showed inconsistency across the cuticle layer, hypodermal areas, and distal gonad. These findings confirm the inhibition of ATGL-1’s lipolytic activity in diabesity-induced worms (**Figure 6D**).

## Discussion

Decades of research on diabesity have unveiled the central players participating as age-related regulatory pathways in *C. elegans*. However, the specific role of lipid droplets in governing lifespan and metabolic disorders has remained elusive. Initial findings have indicated a complex and intricate pathophysiological reaction in *C. elegans* triggered by the supplementation of high glucose and cholesterol. This supplementation has led to conditions such as obesity, hyperglycaemia, as well as collagen and glycation impairment – the classic indicators of metabolic disorders, particularly in diabesity. *C. elegans* serves as an appropriate model organism for studying human metabolic disorders, including obesity (12, 78, 79). Given the similarity between *C. elegans* lipid and carbohydrate metabolism and those observed in mammals, alongside its fully sequenced genome, this tiny nematode serves as an ideal *in vivo* model for studying diabesity. High levels of glucose and cholesterol tend to disrupt cellular homeostasis, increase oxidative stress, and shorten lifespan in organisms such as mice and drosophila (80). In this study, the introduction of high levels of glucose and cholesterol shortened the lifespan of *C. elegans* (**Figure 1A**), increased its body length, compromised locomotion, and affected pharyngeal pumping (**Figures 1C, D**). These outcomes highlight the adverse effects of over-nutrition on the organism’s health. Diets high in glucose have been shown to increase fat deposition and TAG storage in rodents, fruit flies, and zebrafish, mirroring observations in worms. This research aids in understanding metabolic diseases and the impact of dietary choices on health (81). Supplementation of NGM medium with high percentage of glucose resulted in increased fat deposition and TAG storage in worms, revealing heightened variability in comparison to the control group (**Figures 1B**, **2E**).

Elevated ROS production appears to play a significant role in the aging process (82). Lipofuscin, an indicator of both oxidative stress and aging in *C. eleg*ans, is an auto fluorescent pigment that gradually accumulates over time (83), particularly within lysosomes and gut granules of the intestine (84). In our study a higher level of ROS was observed in the diabesity condition. This aligns with reports suggesting that metabolic overload associated with obesity and T2DM, characterized by elevated levels of glucose and free fatty acids (FFA), leads to increased intracellular accumulation of ROS in models such as mice also showed heightened oxidative stress and accumulation of lipofuscin when glucose levels were elevated (85). In our immunofluorescence assay, we observed increased lipofuscin accumulation following glucose treatment, consistent with the observed generation of ROS (**Figures 3A-C**) The TAGs constitute the primary component of lipid droplets, serving as efficient energy reservoirs within *C. elegans* (86, 87). The control mechanisms governing lipid storage have been discerned to revolve around lipid droplets, a phenomenon intertwined with significant metabolic disorders such as diabetes and obesity. Notably, the emergence of insulin resistance in *C. elegans* appears to occur through mechanisms that are conserved across evolution. This observation is underscored by the resemblance in transcriptional responses triggered by elevated levels of glucose and lipids, which involve analogous insulin/IGF-1-like signaling pathways found in humans (88). The proteomic data in the present study showed the insulin-like receptor pathway is among one which has activated in diabesity animals; this is central pathways involved in life span determination. The association between type 2 diabetes and various neurodegenerative conditions has been extensively reported (89–91). Previous studies have indicated that individuals with high glycemic indexes are more susceptible to developing these diseases, highlighting a probable prevalence of insulin resistance among these patients (92). According to (15), a mechanism wherein a high-glucose diet triggers DAF-2-independent regulation of lifespan in *C. elegans*. This independent regulation of body movement (**Figures 4A, B**) and increased body size in a DAF-independent manner bolsters the notion of alternative pathways being activated by high concentrations of glucose.

The interactions between lipid metabolisms highlight the pivotal role of lipid metabolism in maintaining tissue integrity and cellular homeostasis. LD formation and turnover are closely regulated by factors such as hormones (like insulin), dietary lipids, and metabolic conditions (such as fasting versus feeding) in organisms such as mice and Drosophila (93). The interplay becomes particularly evident through the formation of lipid droplets (**Figures 2A-C**), primilarly via *de novo* synthesis within the ER (94). A recent investigation has elucidated the presence of nuclear fat in intestinal and adipose tissues, orchestrating various facets of their physiological functions in *C. elegans* (95). The gene ontology analyses conducted in this study have provided insights into the differential regulation of these specific pathways (**Figures 5A-E**). Furthermore, the protein-protein interaction map has illuminated the complex interrelationships among the previously mentioned proteins, emphasizing their pivotal roles in diverse metabolic, structural, and cellular functions. Overall, the findings obtained from proteomics and the subsequent validation experiments suggest a hypothesis that the expression of several crucial proteins, particularly those involved in the insulin/IGF- like signaling pathway, glycolysis, metabolic process, and cell adhesion, were subject to regulation that potentially contributes to the development of diabesity (96).

**Figure 7:**
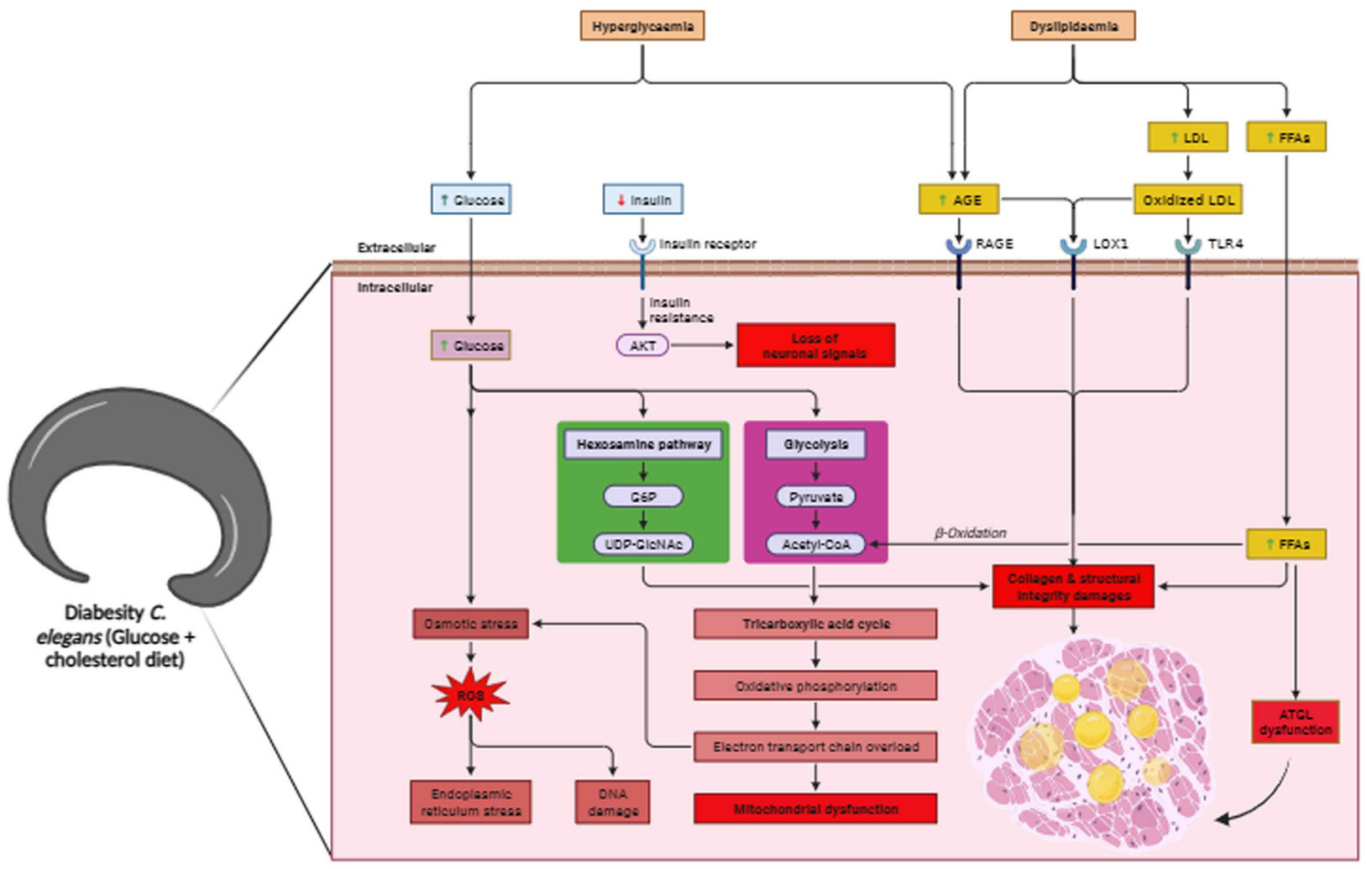
Overall schematic representation of diabesity in *C. elegans*. ATGL dysfunction in diabesity leads to a combination of hyperglycemia and dyslipidemia, resulting in the loss of neuronal signals, increased oxidative stress, and advanced glycation end-products. These disruptions in metabolism elevate free fatty acids, leading to higher lipid accumulation. Concurrently, impaired lipid metabolism and ATGL dysfunction induce endoplasmic reticulum (ER) stress, compromising structural integrity, particularly collagen. The supplementation of glucose with cholesterol-rich diets collectively reduces lifespan by accelerating both glucotoxicity and lipotoxicity, culminating in mitochondrial dysfunction.

The presence of a hypoglycaemic and hypolipidemic environment induces the formation of disorganized collagen fibrils due to the presence of AGE products, leading to reduced skin elasticity, diminished thickness, collagen denaturation, and altered plasticity (97). Indeed, the results of the present study are in line with recent research that highlights the connection between diabetes, obesity, and disruptions in collagen structure and metabolism. Accumulations of over-glycosylated collagens and alterations in the concentrations of sulphated proteoglycan have been implicated in these conditions. These changes in collagen composition and proteoglycan concentrations can lead to morphological alterations such as basement membrane thickening (**Figures 6A, B**). This underscores the complex interplay between metabolic disorders, such as diabetes and obesity, and their impact on the structural integrity of tissues, particularly collagen-rich structures (98). Notably, AGEs form through a non-enzymatic reaction between sugars and proteins/lipids, accumulating in tissues, especially in diabetes, leading to reduced collagen production and structural disruptions. AGEs can cross-link collagen fibers, altering their structure and function. This cross-linking process, known as glycation, stiffens collagen and reduces its flexibility. It contributes to the pathological changes observed in tissues affected by diabetes, such as vascular complications and skin aging (98, 99). ATGL expression serves as a regulatory element in the balance of glucose and lipid metabolism, playing a significant part in the development of lipotoxicity during diabesity in *C. elegans*. Reports consistently validate that lipolysis, a pivotal aspect of fat metabolism, is regulated by multiple crucial factors. Among these, ATGL-1 stands out as a central enzyme accountable for catalyzing the hydrolysis of triglycerides (TAGs) into fatty acids (76). Current studies affirm ATGL-1’s role in diabesity as a key factor in lipolysis regulation. Deactivating ATGL-1 doesn’t promote longevity in diabesity, suggesting its turnover function. It’s transcriptionally regulated, preserving lipid droplet homeostasis in nuclear compartments with aging, reinforcing its pivotal role in diabesity-related lipolysis regulation (100). Notably, the mitigation of lipotoxic lipid accumulation hinges on the activity of the triglyceride lipase ATGL-1 within the intestinal nuclei. Further corroborating the pivotal role of ATGL-1 in organismal equilibrium and tissue functioning, its upregulation effectively sustains LDs and even contributes to an extension of lifespan (101). ATGL-1 deficiency leads to the enlargement of LDs within the intestinal cells of diabesity-affected *C. elegans* when compared to the control. Moreover, immunofluorescence findings revealed that ATGL was primarily concentrated in the epidermal region. It was inconsistently present in the cuticle layer, distinct hypodermal regions, and the distal gonad at endogenous levels in control comparison to diabesity (**Figures 6C, D**). The intricate link between ATGL-1 deficiency, LD morphology, and diabesity’s metabolic disruptions is highlighted here. These findings reinforce the crucial role of lipid-droplet-mediated protein degradation in longevity and aging. They support the hypothesis that lipid droplets maintain cellular balance by stabilizing proteomes and organizing protein degradation machinery, suggesting their pivotal role in cellular homeostasis. In conclusion this study highlights ATGL’s significant impact on protein function, influencing both lipid and glucose processes, and upregulating genes associated with glucose metabolism (**Figure 7**). In conditions like diabesity, inhibiting ATGL disrupts glycolysis, lipid formation, fatty acid oxidation, and influences aging, all processes reliant on ATGL. This positions ATGL as a crucial contributor in diabesity within *C. elegans*, potentially serving as a biomarker. However, a more comprehensive understanding of ATGL’s specific role within the metabolic framework of diabesity necessitates further investigation.

## Data Availability

The datasets presented in this study can be found in online repositories. The names of the repository/repositories and accession number(s) can be found below: https://www.ebi.ac.uk/pride/, accession number: PXD057033.

## Supplementary material

Supplementary Figure 1 Analysis of lipids using thin layer chromatography of control and diabesity protein along with standard.

Supplementary Table 1 List of down regulated proteins present in Control and Diabesity animals identified using LC-MS.

Supplementary Table 2 List of up regulated proteins present in Control and Diabesity animals identified using LC-MS.

Supplementary Table 3 Chemical list used for the study.
