# Supplementary material for: Exploring diabesity pathophysiology through proteomic analysis using Caenorhabditis elegans
Source: Front Endocrinol (Lausanne). 2024 Oct 30;15:1383520. doi: 10.3389/fendo.2024.1383520 (PMC11557309; doi:10.3389/fendo.2024.1383520)
Supplement: Supplementary Figure 1 — Analysis of lipids using thin layer chromatography of control and diabesity protein along with standard. [file DataSheet1.pdf]

Supplementary Figures

Figure S1

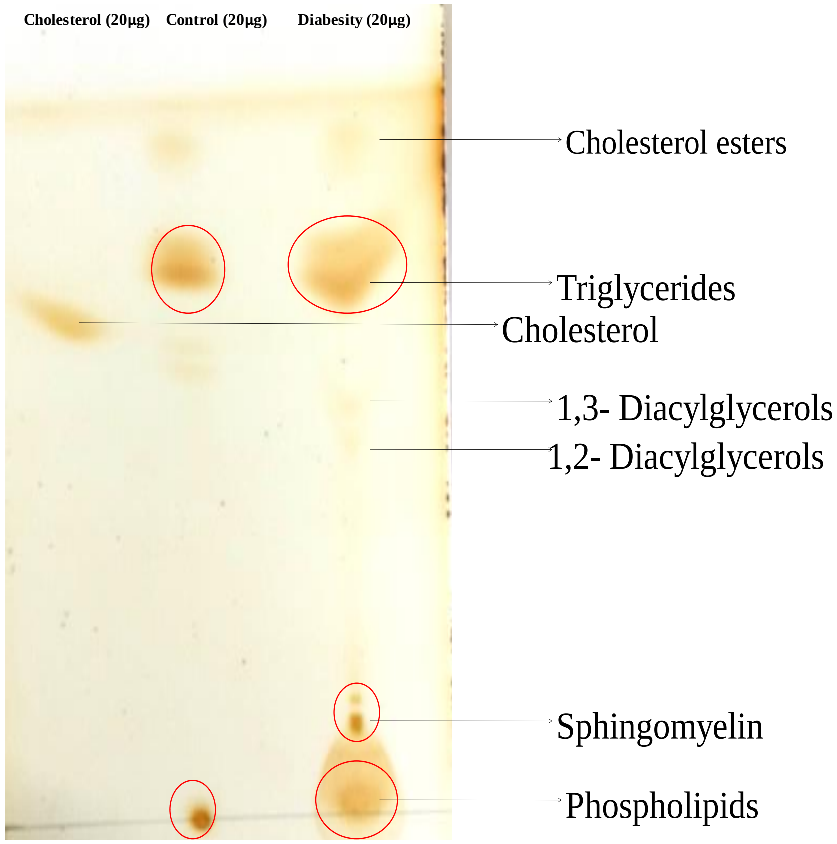

Figure S1: Analysis of lipids using thin layer chromatography of control and diabesity protein along with standard.

Supplementary Tables

Table S1: List of down regulated proteins present in Control and Diabesity animals identified using LC-MS.

S.NO	PROTEIN NAME	GENE NAME	FOLD CHANGE	ANOVA
**1.**	Mesocentin	*dig-1*	0.166	0.001
**2.**	VWFA domain-containing protein	*vwa-8*	0.2	0.001
**3.**	Non- centrosomal microtubule array protein 1	*noca-1*	0.125	0.001
**4.**	RRM domain containing protein	CELE_ZK856.18 ZK856.18	0.587	0.001
**5.**	Patanin like phospholipase domain containing protein	*atgl-1*	0.5414	0.0001
**6.**	Collagen	*col-72*	0.27	0.001
**7.**	Cuticle collagen 19	*col-19*	0.587	0.001
**8.**	Putative extracellular sulfatase Sulf-1 homolog	*sul-1*	0.142	0.005
**9.**	Vitellogenin-5	*vit-5*	0.47	0.05
**10.**	Vitellogenin-3	*vit-3*	0.38	0.02

Table S2: List of up regulated proteins present in Control and Diabesity animals identified using LC-MS.

S.NO	PROTEIN NAME	GENE NAME	FOLD CHANGE	ANOVA
**1.**	Acyl_transf_3 domain containing protein	*oac-11*	2.15	0.001
**2.**	7TM GPCR domain containing protein	*srx-3*	1.83	0.03
**3.**	Fatty acid synthase	*pks-1*	2.5	0.04
**4.**	DNA-directed RNA polymerase II subunit RPB1	*rpb-1*	1.57	0.04
**5.**	Phosphoenol pyruvate carboxy kinase (GTP)	*pck-2*	2.25	0.005
**6.**	Mitogen-activated protein kinase	*mapk-15*	1.56	0.005
**7.**	Protein O-GlcNAc transferase	*ogt-1*	1.666	0.001
**8.**	Ubiquitin ligase complex subunit	*gid-1*	1.5	0.02

Table S3: Chemical list used for the study

Chemicals and Antibodies	Grade
Glucose	(G8270) Sigma-Aldrich

Cholesterol	(GRM6048) HiMedia
ATGL-1	(sc-365278) Santa Cruz Biotechnology, Inc
Beta-actin	(A1978) Sigma-Aldrich
MTBE	(34875) Sigma-Aldrich
Nile red	(19123) Sigma-Aldrich
DCF-DA	(287810) Sigma-Aldrich
Alexa Fluor™ Plus 488	(A32723) Invitrogen
Merck Silica Gel 60 HPTLC plates	(1.12363) Sigma-Aldrich
